# Editorial: Exercise in aquatic environment and health

**DOI:** 10.3389/fspor.2026.1948082

**Published:** 2026-08-14

**Authors:** Carlos Farinha, Catarina Santos, Hélder Santos, Paula Martinez, Yury Rosales-Ricardo, João Serrano

**Affiliations:** 1 Department of Sports and Well-Being – Polytechnic Institute of Castelo Branco, Castelo Branco, Portugal; 2 SPRINT Sport Physical Activity and Health Research & Innovation Center, Castelo Branco, Portugal; 3 Department of Sport Sciences, Exercise and Health, University of Trás-os-Montes and Alto Douro, Vila Real, Portugal; 4 Higher Education School, Polytechnic of Coimbra, Coimbra, Portugal; 5 SPRINT Sport Physical Activity and Health Research & Innovation Center, Coimbra, Portugal; 6 Coimbra School of Health Technology—IPC, Coimbra, Portugal; 7 Federal University of Mato Grosso do Sul, Campo Grande, Brazil; 8 Universidad Tecnológica del Perú, Lima, Peru

**Keywords:** aging, aquatic exercise, aquatic rehabilitation, health, hydro gymnastics, hydrotherapy, water fitness

Physical exercise in aquatic environments has gained increasing importance in promoting health, rehabilitating patients, facilitating motor learning, and preventing functional limitations across different populations. The specific properties of water, such as buoyancy, hydrostatic pressure, multidirectional resistance, and the possibility of temperature control, make it a particularly relevant context for individuals with varying levels of physical fitness, functional limitations, fear of falling, pain, or the need for exercise programmes with lower joint impact. In this sense, this Research Topic aims to address crucial issues, ranging from optimizing different protocols for aquatic exercise at different water temperatures to gaining a deeper understanding of its effects on health.

Seven articles were included in this Special Issue, comprising five original research articles (Kwok et al.; Varveri et al.; Alkasasbeh et al.; Bevins et al.; Ramos et al.) and two systematic reviews (Gao et al.; Mańko et al.). The main areas of interest in these studies were aging, the clinical context, metabolic responses, learning, and risks associated with the swimming pool environment.

In the field of ageing, Gao et al. conducted a systematic review with meta-analysis of randomized controlled trials on the effects of aquatic exercise in older adults. The authors observed improvements in muscle strength, flexibility, functional mobility, body fat percentage, and total cholesterol, suggesting that aquatic exercise may contribute to maintaining functional independence and improving certain indicators of metabolic health in this population. Similarly, Mańko et al. analyzed the effectiveness of aquatic Ai Chi on balance indicators in older adults. The review included seven studies and concluded that aquatic Ai Chi appears to improve static and dynamic balance, with a potential application in fall prevention and as an adjunct to geriatric physiotherapy.

The application of aquatic exercise in clinical contexts was explored by Bevins et al., who assessed the feasibility of an aquatic treadmill training program for community-dwelling stroke survivors in a pilot study. Despite the small sample size, high adherence, an absence of adverse events, and preliminary improvements in indicators of cerebrovascular function and gait were observed. These findings suggest that aquatic treadmill training could represent a promising strategy in post-stroke rehabilitation and should be investigated in randomized controlled trials with more robust sample sizes.

Regarding metabolic responses to exercise, Kwok et al. compared the acute effects of aquatic high-intensity interval training with and without additional resistance in healthy adults. Metabolic and cardiorespiratory responses were similar between protocols, suggesting that different forms of aquatic interval training can be used to achieve comparable physiological goals, with the potential to diversify exercise prescriptions for healthy adults.

Learning, aquatic competence, and safety were also addressed in this Research Topic. Varveri et al. demonstrated that aquatic readiness, understood as the ability to adapt and function in the aquatic environment, can be improved through systematic training. Although pure swimming training produced improvements, the specific aquatic adaptation program yielded greater gains in healthy adolescents, reinforcing the importance of exercises focused on breathing control, sensory adaptation, confidence, and safety in the water. In turn, Alkasasbeh et al. investigated the impact of a mobile application that supports swimming learning among university students. The use of the application did not alter intrinsic motivation but reduced fear of water, suggesting that digital tools could complement face-to-face teaching, particularly when psychological barriers to learning are present.

In addition to the benefits of aquatic exercise, this Research topic also included an important perspective on the risks associated with the swimming pool environment. Ramos et al. analyzed allergy-compatible symptoms in Portuguese federated swimmers using the AQUA® questionnaire. The authors found a high prevalence of positive screenings, particularly among younger swimmers, women, athletes with a weekly training frequency of >7 sessions/week, and individuals exposed to water temperatures above 25 °C. These findings reinforce the need to monitor respiratory and allergic symptoms, along with the importance of appropriate management of ventilation, temperature, and air quality in swimming pools.

Taken together, the studies published in this Research Topic show that exercise in aquatic environments constitutes a multidimensional intervention, with implications for public health, rehabilitation, active aging, swimming pedagogy, aquatic safety, and functional performance. The contributions gathered here demonstrate that the aquatic environment should not be merely viewed as an alternative to land-based exercise, but rather as a context with its own specific characteristics, capable of generating specific responses and adapting to different populations and objectives.

Despite the presented advances, relevant gaps remain. Future research should include larger samples, longitudinal designs, randomized controlled trials, more detailed descriptions of exercise protocols, rigorous monitoring of intensity, control of pool environmental characteristics, and direct comparisons between different aquatic and land-based programs. It will also be important to further explore the role of water temperature, digital technologies, long-term adherence, and the physiological, psychological, and neuromotor mechanisms associated with the benefits of aquatic exercise. Thus, this Research Topic contributes to consolidating exercise in aquatic environments as a relevant, safe, and adaptable strategy for health promotion, prevention, learning, and rehabilitation throughout the life course.

