# Correction: Editorial: Exercise in aquatic environment and health

**DOI:** 10.3389/fspor.2026.1979752

**Published:** 2026-09-07

**Authors:** Carlos Farinha, Catarina Santos, Hélder Santos, Paula Martinez, Yury Rosales-Ricardo, João Serrano

**Affiliations:** 1 Department of Sports and Well-Being – Polytechnic Institute of Castelo Branco, Castelo Branco, Portugal; 2 SPRINT Sport Physical Activity and Health Research & Innovation Center, Castelo Branco, Portugal; 3 Department of Sport Sciences, Exercise and Health, University of Trás-os-Montes and Alto Douro, Vila Real, Portugal; 4 Higher Education School, Polytechnic of Coimbra, Coimbra, Portugal; 5 SPRINT Sport Physical Activity and Health Research & Innovation Center, Coimbra, Portugal; 6 Coimbra School of Health Technology—IPC, Coimbra, Portugal; 7 Federal University of Mato Grosso do Sul, Campo Grande, Brazil; 8 Universidad Tecnológica del Perú, Lima, Peru

**Keywords:** aging, aquatic exercise, aquatic rehabilitation, health, hydro gymnastics, hydrotherapy, water fitness

The author name Yury Rosales-Ricardo was erroneously written as Yury Ricardo.

Affiliation “Universidad Tecnológica del Perú, Lima, Peru” was omitted from the paper. This affiliation has now been added for author Yury Rosales-Ricardo.

Affiliation “University San Gregorio de Portoviejo, Portoviejo, Ecuador” was erroneously included in the paper. This affiliation has now been removed for author Yury Rosales-Ricardo.

The original version of this article has been updated.

